# Acute neurotoxicity in a child following multi-component medicinal fungi supplementation: a case report

**DOI:** 10.1186/s12906-026-05367-6

**Published:** 2026-04-30

**Authors:** Hacer Efnan Melek Arsoy, Öner Özdemi̇r

**Affiliations:** 1 Department of Pediatrics, Faculty of Medicine, Sakarya University, Sakarya, Türkiye; 2 Division of Allergy and Immunology, Department of Pediatrics, Faculty of Medicine, Sakarya University, Sakarya, Türkiye

**Keywords:** Ganoderma lucidum, Reishi mushroom, Hallucination, Drug intoxication

## Abstract

**Background:**

Medicinal fungi supplements have gained widespread recognition, increasing off-label use in pediatric populations. However, their safety profile remains inadequately characterized, and the potential for toxic effects, including neurotoxicity, is underrecognized in clinical practice. Here we report a pediatric case of suspected multi-component medicinal fungi supplement-related neurotoxicity, in which the parents were aware that the ingredient was a mushroom for their son’s alternative treatment.

**Case presentation:**

We report acute neurotoxicity in a 9-year-old boy with prematurity, cerebral palsy, and well-controlled epilepsy presenting with generalized myoclonic seizures, hallucinations, and altered mental status following ingestion of multiple medicinal fungi supplements, including *Ganoderma lucidum* and *Cordyceps sinensis*. Comprehensive investigations, including laboratory studies and neuroimaging, excluded infectious, structural, and metabolic etiologies. The temporal relationship between dose escalation of the supplement and symptom onset, combined with resolution upon discontinuation, suggested a supplement-related mechanism.

**Conclusions:**

This case highlights the potential neurotoxic effects of mushroom supplements containing both *Ganoderma lucidum* (reishi mushroom) and *Cordyceps sinensis* in children with underlying neurological disorders. The temporal association between dose escalation and symptom onset, with complete symptom resolution upon discontinuation, suggests a supplement-related mechanism. Given the increasing use of medicinal fungi in complementary medicine, greater clinical vigilance and public awareness of their potential risks are essential.

**Supplementary Information:**

The online version contains supplementary material available at 10.1186/s12906-026-05367-6.

## Introduction

Medicinal fungi supplements have been widely used in traditional medicine for their purported immunomodulatory, anti-inflammatory, and neuroprotective properties [1]. However, their safety profile remains inadequately characterized, particularly in pediatric populations. In recent years, administration of *Ganoderma lucidum (G.lucidum)* and *Cordyceps sinensis (C. sinensis)* supplements has increased [2, 3].

Despite its widespread perception as a safe natural supplement, potential toxicities remain underrecognized. Literature review reveals no well-documented pediatric cases of acute neurotoxicity clearly attributable to medicinal fungi supplements. Published safety concerns instead focus primarily on hepatotoxicity and hematologic or immune effects, mostly in adults [4–6]. We present a unique pediatric case of acute neurotoxicity following reishi-containing supplement ingestion, aiming to raise awareness of potential neurological adverse effects.

## Case presentation

A 9-year-old male with prematurity, cerebral palsy, and epilepsy presented with generalized seizures, visual hallucinations, and altered mental status. He exhibited progressive confusion, agitation, and severe restlessness, and a 48 h of sleep disturbances. Before admission, the patient had experienced widespread intermittent myoclonic jerks accompanied by sudden, repetitive vocalizations for three hours without an identifiable trigger. There was no history of fever, infection, recent trauma, or concurrent medications other than levetiracetam. The patient had been on stable antiepileptic therapy with Levetiracetam (15 mg/kg per day) for over five years without any breakthrough seizures, and his last documented short (1–2 min) tonic episode occurred two years earlier.

Physical examination showed appropriate growth (height 30th, weight 15th percentile) and adequate nutrition. He was afebrile (36.8 °C), tachycardic (125/min), and normotensive (110/85 mmHg, ~ 50–75th percentile). Neurological assessment revealed agitation and hallucinations without new focal deficits, beyond baseline developmental delay. Laboratory investigations—including a complete blood count, serum electrolytes, liver and renal function tests, and inflammatory markers—were all within normal limits. Urine and serum toxicology screens were negative. Cranial computed tomography showed no acute abnormalities. Electroencephalography (EEG), performed 10 h after admission, showed no epileptiform activity or findings suggestive of an ictal or postictal state. An electrocardiogram showed sinus tachycardia.

On day 2, the parents disclosed that they had been administering multiple medicinal fungi supplements for 3–4 months, including *G. lucidum* powder (850 mg/day), *C. sinensis* extract combined with *G. lucidum* (275 mg/day), and a mixed coffee product containing *G. Lucidum* obtained from an online health supplement retailer. They had doubled the total dosage one week before symptom onset without medical advice.

The patient received supportive care only, without pharmacological intervention. Neurological symptoms completely resolved on day 2 following cessation of supplement use. He was discharged on day 3 with complete recovery and explicit counseling to avoid further use.

## Discussion

### Potential mechanisms of neurotoxicity

Medicinal fungi products, such as G. lucidum and C. sinensis are widely used for their immunomodulatory, anti-inflammatory, and neuroprotective properties [6]. These fungi contain a variety of bioactive compounds, including polysaccharides, triterpenoids, and nucleoside derivatives, some of which are capable of interacting with neuronal signaling pathways and influencing neuronal physiology [7–9]. Although experimental studies suggest potential neuromodulatory activity, clinical safety data—particularly in pediatric populations—remain limited. Therefore, idiosyncratic reactions, supplement contamination, or dose-related toxicity cannot be excluded when unexplained neurological symptoms occur following ingestion of such products [4, 9, 10].

Animal studies indicate that *G. lucidum* derivatives influence dopaminergic and serotonergic neurotransmission, which may explain psychiatric manifestations including agitation and hallucinations [11, 12]. Similarly, *C. sinensis* can produce bioactive metabolites that may interact with other chemical constituents, potentially enhancing neurotoxicity [13, 14]. Synergistic or additive effects between these compounds may have contributed to the observed neurotoxicity in our case.

Children’s immature hepatic and renal metabolism predispose them to accumulate such compounds, and preexisting neurological disorders may further increase vulnerability due to impaired blood–brain barrier integrity. These mechanisms provide a plausible explanation for the acute neurotoxicity observed in our patient [15, 16].

### Exclusion of alternative causes

Comprehensive evaluation excluded infectious, metabolic, and structural etiologies. EEG showed no epileptiform activity, ruling out ictal or postictal phenomena. Visual hallucinations and behavioral changes differed markedly from prior seizure semiology. Although the patient’s underlying epilepsy and cerebral palsy could theoretically predispose to seizure recurrence, the two-year stable course before supplement exposure and the dramatic new symptom pattern strongly argue against disease progression and favor an acute toxic event. The temporal relationship between supplement dose escalation and symptom onset is striking. Acute neuropsychiatric symptoms emerged within one week of supplement dosage doubling and resolved within 24 h of cessation. This sequence strongly suggests a causal relationship between supplement exposure and the acute neurological presentation.

Levetiracetam-induced neurotoxicity is unlikely: (1) stable dosing for five years without prior behavioral effects; (2) no recent dose escalation; (3) normal electroencephalography during acute symptoms [17].

### Regulatory and clinical considerations

Our patient was exposed to three medicinal fungi preparations. The lack of standardized labeling and compositional data hindered the identification of the toxic agent. Possible explanations include: (1) *G. lucidum* alone caused toxicity; (2) synergistic effects between *G. lucidum* and *C. sinensis;* or (3) an unknown constituent triggered toxicity. This regulatory gap, including a lack of standardized labeling and quality control, prevents accurate causal determination [18]. However, we lacked serum biomarker quantification for reishi or cordyceps constituents, reflecting the acute clinical context and absence of standardized pediatric toxicology protocols. Future cases should include targeted biomarker testing [19].

### Clinical and public health implications

This case underscores the importance of routinely screening for complementary and alternative medicine use when evaluating pediatric neurological emergencies. Physicians should specifically inquire about herbal or mushroom-based products, recent dose escalations, and unregulated supplements. Healthcare providers should warn families that “natural” is not safe for children.

Future studies should aim to establish pharmacokinetic data, toxic dose thresholds, and pediatric safety profiles for *G. lucidum* and *C. sinensis* [16, 18, 20]. Collaboration between clinicians, toxicologists, and regulatory agencies will be vital to improve public awareness and implement safety guidelines for pediatric supplement use.

### Limitations

The primary limitation was the inability to perform detailed toxicological analysis of the supplement samples for adulterants or contaminants. Although levetiracetam is known for its predictable pharmacokinetics and minimal drug-drug interactions, serum drug levels were not measured during the acute phase; therefore, a pharmacokinetic interaction cannot be definitively excluded. Concurrent exposure to multiple preparations prevented identification of a single agent, and long-term neurodevelopmental follow-up data were unavailable. As a single case report, causality cannot be definitively established.

## Conclusion

Healthcare providers should maintain heightened vigilance regarding herbal and dietary supplement use in children presenting with unexplained neurological symptoms. This case highlights the need for improved regulation and pediatric safety evaluation of medicinal fungi supplements. Routine screening for supplement use and education of families regarding risks associated with unregulated herbal products are essential for comprehensive pediatric neurological care. Future studies should establish pharmacokinetic and safety profiles of medicinal fungi supplements in children.

### Take-home message

1. *G.lucidum and C. sinensis* products lack standardized pediatric dosage and regulatory control.
2. Herbal supplement use should be considered in the differential diagnosis of unexplained neurological symptoms.
3. Public awareness of the potential risks of unregulated herbal products must be increased.

## Supplementary Information

Supplementary Material 1.

## Data Availability

Patient data are available from the corresponding author upon reasonable request. (hacermelekarsoy@sakarya.edu.tr).

## Abbreviations

EEG: Electroencephalography
CNS: Central nervous system
