# Supplementary material for: Acute neurotoxicity in a child following multi-component medicinal fungi supplementation: a case report
Source: BMC Complement Med Ther. 2026 Apr 30;26:214. doi: 10.1186/s12906-026-05367-6 (PMC13281238; doi:10.1186/s12906-026-05367-6)
Supplement: Supplementary file 1 — Supplementary Material 1. [file 12906_2026_5367_MOESM1_ESM.pdf]

# CARE Checklist of information to include when writing a case report

CARE case report guidelines

(cc) BY-NC-ND

| Topic | Item | Checklist item description | Reported on Line |
|---|---|---|---|
| **Title** | 1 | The diagnosis or intervention of primary focus followed by the words “case report” | Title page, line 1._______ |
| **Key Words** | 2 | 2 to 5 key words that identify diagnoses or interventions in this case report, including "case report" | Abstract / Keywords section._ |
| **Abstract (no references)** | 3a | Introduction: What is unique about this case and what does it add to the scientific literature? | Abstract, lines 2–5 |
| | 3b | Main symptoms and/or important clinical findings | Abstract, lines 6–8 |
| | 3c | The main diagnoses, therapeutic interventions, and outcomes. | Abstract, lines 8–15. |
| | 3d | Conclusion—What is the main “take-away” lesson(s) from this case? | Abstract, lines 15–18 |
| **Introduction** | 4 | One or two paragraphs summarizing why this case is unique (**may include** references) | Introduction, paragraph 1 |
| **Patient Information** | 5a | De-identified patient specific information | Case Presentation, lines 1–10 |
| | 5b | Primary concerns and symptoms of the patient | Case Presentation,paragrph2 |
| | 5c | Medical, family, and psycho-social history including relevant genetic information | Case Presentation, paragraph 3 |
| | 5d | Relevant past interventions with outcomes | Case Presentation, paragraph 4 |
| **Clinical Findings** | 6 | Describe significant physical examination (PE) and important clinical findings | Case Presentation, paragraph 5_ |
| **Timeline** | 7 | Historical and current information from this episode of care organized as a timeline | Case Presentation,timelineparag |
| **Diagnostic Assessment** | 8a | Diagnostic testing (such as PE, laboratory testing, imaging, surveys) | Case Presentation,investigations p. |
| | 8b | Diagnostic challenges (such as access to testing, financial, or cultural) | Discussion, section 3 |
| | 8c | Diagnosis (including other diagnoses considered) | Discussion, section 2 |
| | 8d | Prognosis (such as staging in oncology) where applicable | Discussion, follow-up paragraph_ |
| **Therapeutic Intervention** | 9a | Types of therapeutic intervention (such as pharmacologic, surgical, preventive, self-care) | Reported – Case Presentation, management paragraph. |
| | 9b | Administration of therapeutic intervention (such as dosage, strength, duration) | Reported – Case Presentation, management paragraph. |
| **Follow-up and Outcomes** | 9c | Changes in therapeutic intervention (with rationale) | Reported – Case Presentation, follow-up paragraph |
| | 10a | Clinician and patient-assessed outcomes (if available) | Reported – Follow-up and Outcomes section. |
| | 10b | Important follow-up diagnostic and other test results | Reported – Follow-up and Outcomes section. |
| | 10c | Intervention adherence and tolerability (How was this assessed?) | Reported – Follow-up paragraph ______ |
| | 10d | Adverse and unanticipated events | Reported – Discussion, Limitations section |
| **Discussion** | 11a | A scientific discussion of the strengths AND limitations associated with this case report | Reported – Discussion, section 5.______________________ |
| | 11b | Discussion of the relevant medical literature **with references** | Reported – Discussion, section 1–4.____ |

**11c** The scientific rationale for any conclusions (including assessment of possible causes)........................................Reported – Discussion, section 2–3.____

**11d** The primary "take-away" lessons of this case report (without references) in a one paragraph conclusion.................Reported – Conclusion / Take-home message.______

**Patient Perspective** **12** The patient should share their perspective in one to two paragraphs on the treatment(s) they received . . ...........Reported – Discussion, final paragraph.________________

**Informed Consent** **13** Did the patient give informed consent? Please provide if requested. Reported – Ethical approval section. . . . . . . . . . . . . . . . . . . . . . . . . . . . . . . . . . . . . .

**Yes X No** ☐

**CARE** case report guidelines

## CARE Checklist of information to include when writing a case report

(cc) BY-NC-ND

✔

| Topic | Item | Checklist item description | Reported on Line |
|---|---|---|---|
| **Title** | **1** | The diagnosis or intervention of primary focus followed by the words "case report" . . . . . . . . . . . . . . . . <br>*Acute Neurotoxicity in a Child after Medicinal Fungi Supplementation: Suspected Role of Ganoderma lucidum*. The title includes the diagnosis/intervention of primary focus and the term "case report'' | |
| **Key Words** | **2** | 2 to 5 key words that identify diagnoses or interventions in this case report, including "case report" <br>Ganoderma lucidum, reishi mushroom, hallucination, drug intoxication | |
| **Abstract (no references)** | **3a** | Introduction: What is unique about this case and what does it add to the scientific literature? <br>This case is unique as it describes acute neurotoxicity associated with Ganoderma lucidum and Cordyceps sinensis supplement use in a pediatric patient—an association rarely documented in the literature. | |
| | **3b** | Main symptoms and/or important clinical findings <br>A 9-year-old boy presented with generalized myoclonic seizures, hallucinations, agitation, and altered mental status. | |
| | **3c** | The main diagnoses, therapeutic interventions, and outcomes: <br>After exclusion of metabolic, structural, and infectious etiologies, neurotoxicity related to multiple medicinal fungi supplements was suspected. The patient received supportive care only; all neurological symptoms resolved within 24 hours of discontinuation. | |
| | **3d** | Conclusion—What is the main "take-away" lesson(s) from this case? <br>Medicinal mushroom supplements, even when marketed as natural, may cause neurotoxic reactions in children. Clinicians should routinely inquire about complementary and alternative medicine use. | |
| **Introduction** | **4** | One or two paragraphs summarizing why this case is unique (**may include** reference**s**) <br>Summarizes the rarity of pediatric neurotoxicity due to suspected Ganoderma lucidum and Cordyceps sinensis, highlighting the novelty of this report. | |
| **Patient Information** | **5a** | De-identified patient specific information <br>A 9-year-old male child; de-identified, no personal identifiers included. | |
| | **5b** | Primary concerns and symptoms of the patient <br>Confusion, agitation, hallucinations, generalized myoclonic seizures, and disturbed sleep. | |
| | **5c** | Medical, family, and psycho-social history including relevant genetic information | |

History of prematurity, cerebral palsy, and well-controlled epilepsy. No relevant family history or psychosocial stressors

**5d** Relevant past interventions with outcomes

On levetiracetam 15 mg/kg/day for 5 years with no breakthrough seizures; last seizure two years prior to this event.

**Clinical Findings**

**6** Describe significant physical examination (PE) and important clinical findings

On examination: afebrile (36.8°C), tachycardic (125 bpm), normotensive (110/85 mmHg), agitation, fluctuating mental status, and visual hallucinations. No new focal neurological deficits beyond baseline.

**Timeline**

**7** Historical and current information from this episode of care organized as a timeline

| Timeframe | Event |
|---|---|
| 3–4 months before admission | Began medicinal fungi supplementation (*G. lucidum* 850 mg/day, *C. sinensis* extract with *G. lucidum* 275 mg/day). |
| 1 week before admission | Parents doubled total dosage without medical consultation. |
| Day 0 | Developed agitation, confusion, visual hallucinations, and seizures. |
| Day 1 | Hospital admission; supportive management started. |
| Day 2 | Symptoms resolved after discontinuation of supplements. |
| Day 3 | Discharged with full recovery and counseling. |

**Diagnostic Assessment**

**8a** Diagnostic testing (such as PE, laboratory testing, imaging, surveys).

CBC, electrolytes, liver/renal tests, inflammatory markers, urine/blood toxicology, drug level, cranial CT, EEG — all within normal limits.

**8b** Diagnostic challenges (such as access to testing, financial, or cultural).

Unavailability of serum biomarkers for *G. lucidum* and *C. sinensis* metabolites; lack of standardized supplement labeling hindered precise identification of toxic components

**8c** Diagnosis (including other diagnoses considered).

Acute neurotoxicity secondary to *Ganoderma lucidum* and *Cordyceps sinensis* supplement ingestion. Other causes (infection, metabolic, epileptic) excluded.

**8d** Prognosis (such as staging in oncology) where applicable

Excellent; complete neurological recovery after discontinuation.

**Therapeutic Intervention**

**9a** Types of therapeutic intervention (such as pharmacologic, surgical, preventive, self-care)

Supportive management; discontinuation of all mushroom supplements.

**9b** Administration of therapeutic intervention (such as dosage, strength, duration)

No pharmacologic treatment administered; hydration and observation only.

**9c** Changes in therapeutic intervention (with rationale)

Cessation of supplements led to complete symptom resolution within 24 hours.

**Follow-up and Outcomes**

**10a** Clinician and patient-assessed outcomes (if available)

Improvement of mental status, return to previous state; no recurrence of seizures.

**10b** Important follow-up diagnostic and other test results without adverse effects.

Normal neurological examination; no residual symptoms.

**10c** Intervention adherence and tolerability (How was this assessed?)

Patient tolerated withdrawal and supportive management

**10d** Adverse and unanticipated events

No further adverse or unanticipated events observed after discharge.

**Discussion**

**11a** A scientific discussion of the strengths AND limitations associated with this case report

Strengths: First pediatric report linking *G. lucidum–C. sinensis* to acute neurotoxicity; comprehensive diagnostic exclusion.
Limitations: No toxicological biomarker analysis; simultaneous use of multiple supplements; retrospective dosage reporting.

**11b** Discussion of the relevant medical literature **with references**....................................................................................

Under each heading, a discussion section is written with valid references on the relevant subject ............................

**11c** The scientific rationale for any conclusions (including assessment of possible causes)

Temporal relationship, symptom pattern, and rapid resolution after supplement cessation provide strong circumstantial evidence of supplement-related neurotoxicity.

**11d** The primary "take-away" lessons of this case report (without references) in a one paragraph conclusion

Natural does not mean safe. Clinicians should always ask about supplement use in pediatric neurological emergencies and educate families about unregulated herbal products.

**Patient Perspective**

**12** The patient should share their perspective in one to two paragraphs on the treatment(s) they received

The patient's parents expressed surprise that a "natural" supplement could cause such severe symptoms. They acknowledged the importance of consulting physicians before giving any non-prescribed products and agreed to avoid all herbal or mushroom-based preparations in the future.

**Informed Consent**

**13** Did the patient give informed consent? Please provide if requested **Yes** x☐ **No** ☐

Yes.
Written informed consent for publication was obtained from the patient's parents in accordance with institutional and ethical standards (Declaration of Helsinki).
